# A Study on Environmental, Social and Governance Fund Performance and Fund Flow: Evidence From Korea Stock Exchange

**DOI:** 10.3389/fpsyg.2021.811099

**Published:** 2022-01-31

**Authors:** Dongchul Kwak, Yu Kyum Kim, Il Sook Kwon

**Affiliations:** ^1^Department of Chinese Business and Economics, Hannam University, Daejeon, South Korea; ^2^Department of Global Economics and Commerce, Hannam University, Daejeon, South Korea

**Keywords:** ESG fund, fund flow, fund performance, volatility, asymmetry, propensity score matching

## Abstract

This study analyzed the sensitivity between fund flow and fund performance with Korean funds, whether there would be a difference in the sensitivity between environmental, social and governance (ESG) funds and non-ESG funds, and whether there was a difference in sensitivity according to the type of past fund performance (positive and negative). The main results of the analysis are as follows. First, the analysis of the fund flow–performance correlation of Korean funds revealed that they had a negative (−) correlation and the ESG did not affect fund flow. Analysis of the difference in sensitivity between fund flow and performance volatility revealed that there was a negative (−) correlation regardless of the performance measuring method and ESG. Finally, the comparison of fund flow and performance sensitivity according to the type of past fund performance revealed that despite consistent asymmetry, there was little difference in sensitivity asymmetry between ESG funds and non-ESG funds. The results reveal that, unlike the expectation that investors in Korean ESG funds would focus more on non-financial properties like the purpose of investment than on profit, they attach the same importance to fund performance.

## Introduction

A financial market paradigm change begins with institutional changes. There are many institutional changes currently taking place, one of which is environmental, social and governance (ESG). International interest in ESG investment is increasing as the importance of ESG management is emphasized. Consequently, ESG^1^ is the bond market’s biggest topic.

Recently, major domestic and foreign companies and financial institutions have increased the importance of the issue of ESG bonds. There have been many discussions about the origin and definition of ESG. More specifically, ESG is a concept that began with the fact that non-financial factors, in which we had little interest, might shake the essence of the issue’s subject.

To examine it with stricter criteria, it is defined as bonds issued based on Green Bond Principles (GBP), Social Bond Principles (SBP), and Sustainable Bond Guideline (SBG) announced by the International Capital Market Association (ICMA) and those issued based on The Climate Bond Standards (CBS) announced by the Climate Bond Initiative (CBI).

The concept of ESG that had first exhibited an influence on the stock market is spreading fast in the bond market as well, starting in 2020. The most question and concern is “What is the difference between non-ESG bonds and ESG bonds?” The conclusion is the generalization of new bonds. Finally, with global paradigm change, ESG bonds will become common while non-ESG bonds will not be common anymore. Instead of the decrease in credit spread of ESG bonds or becoming excellent bonds, it is possible to see a phenomenon in which credit spread expands as demand for non-ESG bonds decreases.

ESG investment has evolved into an essential element. The size of the ESG bond issues has increased noticeably at home and abroad. In 2015, there were only $80.7 billion in global ESG bonds. In 2020, there were $789.8 billion in ESG bonds. This was almost a 10-fold increase. The weight of responsible funds considering ESG is still lower for bonds than stocks. However, ESG bond numbers are increasing at a significant rate. Responsible investments, which have been limited to stocks, are made actively through bonds. In the ESG bond market, as well as the issue of bonds to raise specific funds (e.g., green bonds), the method of adjusting the weight of the inclusion in the portfolio considering the ESG performance of the issuer draws attention for the same reasons.

When compared to the global ESG market, the Korean ESG bond market is still in an early stage in terms of size, diversity, and investor base. It needs the power to experience growth. And yet, as the National Pension amended the fund management principle in November 2019, it expanded and applied ESG investment to all asset classes. Hence, Korean investors’ interest in ESG investment has gradually expanded.

This study is primarily concerned with ESG funds. An analysis of the impacts of the setting of ESG on the monetary flow of the Korean funds will be conducted. ESG and non-ESG funds will be compared to examine the relative difference in the sensitivity between fund flow and fund investment performance. The focus will be placed on the method for measuring the fund performance, as well as the type of fund’s past performance. The focus will also be placed on describing the factors with significant positive impacts.

The rest of this article is structured as follows. Section “Literature Review and Hypotheses” summarizes the previous studies of correlations between ESG funds and fund flows. It also presents the empirical hypotheses to be dealt with in this study along with the grounds. Section “Reference Data and Research Methods” describes the characteristics of the samples and the main variables used in the empirical analysis. Section “Result of Empirical Analysis” presents the main results of the empirical analysis, and section “Conclusion” draws a conclusion and provides suggestions.

## Literature Review and Hypotheses

It is difficult to clearly define the concept of ESG investment. It is difficult to accurately classify ESG by subject and to examine the history of ESG investment, its definition differs depending on the culture, religion, values, and belief. By the occasion, it is used in various names, such as Socially Responsible Investment (SRI), Responsible Investment (RI), and ethical investment. The SRI forum in the United States and Europe does not clearly define SRI. Consequently, these terms are used in diverse and mixed ways.

The global ESG investment began from an ethical/religious motivation to exclude specific industries (e.g., alcoholic beverages, tobacco, and weapons manufacturing) in the 1920s. In the 1960s–1970s, responsible investments in the public interest became revitalized. As social interest in global warming, human rights issues, and corporate corruption increased, its meaning and concept evolved. Entering the 1970s, South Africa’s apartheid policy triggered the SRI of institutional investors. In the 1980s, as large accidents took place (e.g., Exxon Valdez oil spill, and Bhopal gas tragedy), environmental issues have drawn more attention. Since 2000, when the Principles for Responsible Investment were enacted by the UN PRI, there have been international public debates. There has also been a revitalization in ESG investments centered around the Pension and Funds Audit Bureau.

The sales of ESG bonds in South Korea began with the issue of green bonds by the Export–Import Bank in 2013. As the demand for investment in ESG bonds increased in the global financial market (e.g., Europe and United States), which set out for expanding investment assets with responsible investment, Korean paper issuers also set out to expand the base of investors through the issue of ESG bonds. The size of the issue of ESG bonds in Won has increased, thanks to the issue of these bonds by many private companies (e.g., POSCO, Hanwha Energy, and Shinhan Financial Group).

ESG bonds began being issued in Won in May of 2018, much later than the ESG bonds that were first issued in foreign currency in 2013. The Korean Development Bank issued green bonds worth 300 billion Won. Later, Shinhan Bank and Korea Southern Power issued green bonds in Won. And in February of 2019, IBK and Woori Bank issued sustainable bonds in Won. Woori Card issued a social bond in Won for the first time as a financial company specializing in the loan business.

The domestic ESG market centered around green bonds in the earlier stage has recently expanded to social bonds and sustainable bonds. In particular, since sustainable bonds can use the funds raised in eco-friendly investments (green bonds) and investments to solve social problems (social bonds), there is an advantage in terms of versatility. Consequently, the size of the issue has tended to expand. In addition, ESG bonds centering around the bonds in a foreign currency (e.g., U.S. Dollars, Euros, and Swiss Francs) are highly preferred by foreign investors in the global market. In 2018, the Korean Development Bank issued bonds in Won. The number of Won-issued bonds has increased in earnest since 2019.

In this investigation, we will examine the advantages and disadvantages of the investors and issuers of ESG bonds, when compared to non-ESG bonds. The investors have advantages (e.g., investment that improves a public utility, opportunities for investment diversification, and easy risk management), since it is possible to check fund uses. In contrast, the disadvantages include low liquidity and the clear performance of cumulative returns. Issuers have advantages (e.g., promotion of an image related to social reliability, security of demands for ESG-related investments in response to the social atmosphere). However, when compared to non-ESG bonds, there are also risks (e.g., additional costs for certification in advance, ex-post facto public notification, and confidence slumps according to non-compliance with the issuance principle).

### Previous Studies

ESG investments emphasize prioritizing the maximization of the return on investment for customers and beneficiaries as a trustee’s duty. The purpose of the investment has been changed from investing in good companies to investing in good companies with bright prospects. The question of whether or not profits can be created in investing in ESG funds continues.

ESG investments aim to increase profitability in priority; however, contributing to social responsibility and capital market fidelity is also a fundamental goal. No conclusions have been drawn concerning whether or not it is possible to achieve non-financial values simultaneously (e.g., ESG values and financial values). Of course, the direction investors want is to consider non-financial values in investments.

There are various opinions in the studies that verified the correlation between ESG investment and financial performance. Bollen (2007) investigated SRI funds, a previous form of ESG funds, and fund flow for the first time and showed that SRI fund flow was more (less) sensitive to positive (negative) time-lag rate of return. The determinants of fund performance and money flow are important topics for fund managers and investors; however, there are differences between non-ESG funds and ESG funds. Chevalier and Ellison (1997) and Sirri and Tufano (1998) noted that the funds with enhanced fund performance had higher money flow; however, there was an asymmetric correlation between fund performance and money flow (Ippolito, 1992; Del Guercio and Tkac, 2002). This asymmetry does not occur in all funds (Del Guercio and Tkac, 2002; James and Karceski, 2006).

According to the result of the survey conducted by Morgan Stanley (2019) (Sustainable Signals), 85% of the institutional investors in asset management companies in the United States responded that non-financial factors were important elements in decision making. Especially, 95% of the millennial generation that would become the mainstream of investment responded that those factors were positive. However, no conclusion has been drawn concerning whether non-financial factors have positive impacts on financial factors. Theoretically, companies in the high ESG class get relatively fewer ESG-related incidents, so the likelihood of exposure to downside risks due to corporate reputation or performance deterioration may decrease. This acts positively on financial performance and returns on investment. On the other hand, the costs that may incur as the investors consider the criteria for ESG investment and decreasing investment opportunities due to the exclusion of items may act negatively on the rate of return.

Friede et al. (2015)^2^ analyzed about 2,000 research papers that described the correlations of ESG factors with companies’ financial performance and reported that 48% concluded the correlation between ESG and financial performance to be positive; 11%, to be negative; and 23%, to be neutral.

**FIGURE 1:**
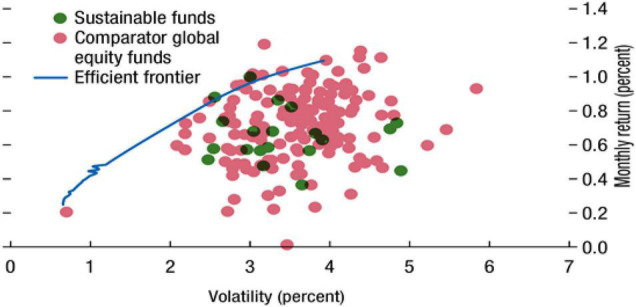
Efficient frontier based on sustainable funds and comparator global equity funds. Source: IMF (Victor Hugo, 2019, p. 10), Global Financial Stability Report.

The IMF Global Financial Stability Report Victor Hugo (2019) shows that there is no consistent evidence that ESG funds have a higher or lower rate of return than non-ESG funds and that the limit of investment in ESG funds leads to a decrease in performance (Figure 1). In addition, Wee et al. (2020) analyzed the correlation between the ESG level of a fund and the fund performance and reported that there was a higher risk-adjusted return in the funds at a high ESG level than in other funds or no statistically significant difference. This means that the investment strategy that attaches importance to ESG factors and reflects them in investment cannot show any decrease in performance. This suggests that in Korea, ESG funds are likely to be “ESG funds in name only.”

In conclusion, there is a lack of ground for judging the performance of ESG investment to be positive or negative. Most countries all over the world have not prepared an environment in which non-financial factors can be measured, and assessment institutions’ appraisal methodologies have not been settled. Especially, since in Korea, ESG investment is in its beginning stage, so it is too early to mention its effects.

### Hypotheses Development

Investors’ choice of funds generally depends on fund performance and risk appetite. In other words, rational investors would compose portfolios based on the past performance of the fund. Thus, rational investors can predict a positive (+) correlation between the past return of the fund and the fund flow (Berk and Green, 2004). In addition, the positive (+) correlation between past performance and fund flow can also be inferred through trading patterns such as a positive feedback strategy (Ippolito, 1992; Gruber, 1996; Sirri and Tufano, 1998; Del Guercio and Tkac, 2002).

However, it is expected that ESG fund investors (e.g., corporate social responsibility activities) would be relatively less sensitive to past performance or volatility (Bollen, 2007; Renneboog et al., 2011).

Hypothesis 1: The fund flow–performance sensitivity would be weaker in ESG funds than in non-ESG funds.

ESG investors decide on investment, actively reflecting non-financial factors such as corporate social responsibility (CSR) activities. Consequently, ESG investors are expected that it would be unlikely that ESG investors decide short-term investment curtailment (withdrawal) even if the financial performance of the fund is poor (Bollen, 2007). Thus, it is assumed that the money flow according to negative investment performance would be less in ESG funds than in non-ESG funds.

Hypothesis 2: The fund flow-negative fund performance sensitivity would be weaker in ESG funds than in non-ESG funds.

There are conflicting claims on the correlation between fund flow and fund performance volatility. According to Busse’s (1999) volatility timing hypothesis, fund managers adjust the market exposure of fund portfolios if the market volatility is expected. Thus, in the funds with low expected performance due to positive feedback trading, there would be a negative (−) correlation between fund performance volatility and fund flow because of fund liquidation or money outflow (Busse, 1999). Meanwhile, noise traders who invest not depending on information become the main cause for letting the market price break away from the fundamental value, and also, because most fund investors are noise traders, their irrational investment behavior (sentiment) may be the main cause for the fluctuation of fund performance (Lee et al., 1991; Black, 1996).

In particular, since positive feedback trading may be accompanied by a short-term volatility increase, the increase in the volatility of the fund performance and fund flow may have a positive (+) correlation.

Hypothesis 3: The fund flow–performance volatility sensitivity would be weaker in ESG funds than in non-ESG funds.

The disposition effect, an irrational investment behavior, may differ depending on the fund performance for several reasons. First, if a positive fund performance is realized, investors would realize the profits (sell-off) earlier as the fund performance volatility increases. Thus, a negative (−) correlation is expected to exist between the fund flow and the volatility. On the contrary, if a negative fund performance is realized, they would keep holding it as the fund performance volatility increases. Thus, a positive (+) correlation is expected between the fund flow and the volatility. Therefore, the correlation between the fund flow and fund performance volatility will appear asymmetric, according to the type of the fund performance. However, the asymmetric sensitivity of the ESG funds would be weaker than that of the non-ESG funds.

Hypothesis 4: The fund flow-volatility sensitivity would be asymmetric according to fund performance, and the asymmetry would be weaker in ESG funds than in non-ESG funds.

## Reference Data and Research Methods

### Reference Data Set

The reference dataset used in this study includes funds classified as ESG funds in the fund-related materials provided by the Korean Fund Ratings (KFR). This information was matched with non-ESG funds with the fund characteristics most similar to them (Korea Fund Ratings [KFR], 2021). The analysis period was 6 years and 7 months (January 2015 to July 2021). The analysis involved examining the status of Korean funds. As of July 2021, the number of ESG funds was 20 and the number of non-ESG funds was 337. The number of ESG funds used in the analysis was 20 and the number of non-ESG funds matched with them was 52. The descriptive statistics are summarized in Table 1.

**TABLE 1:** Descriptive statistics on reference data set.

Panel A: All funds						
**Variable**	***N***	**Mean**	**Std. Dev**	**Min**	**Max**	**Median**
AUM (hundred million)	5,582	216.5	422.0	0.0	4799.2	48.7
Flow	5,582	0.396	8.176	−96.399	462.417	0.261
Raw return	5,582	−0.244	4.965	−74.202	97.391	−0.240
MKT-Adj return	5,582	−1.105	7.574	−80.524	95.751	−0.830
CAPM-Adj return	5,582	−0.258	4.966	−74.218	97.376	−0.254
Raw return volatility	4,521	4.269	2.231	1.123	30.224	3.977
MKT-Adj return volatility	4,521	7.359	2.925	1.595	30.333	6.805
CAPM-Adj return volatility	4,521	4.269	2.231	1.123	30.224	3.977
Age	5,582	13.24	4.84	2.00	23.00	15.00
Remuneration Rate (%)	5,582	1.42	0.39	0.21	2.00	1.53

**Panel B: (ESG) funds**						

**Variable**	***N***	**Mean**	**Std. Dev**	**Min**	**Max**	**Median**
AUM (hundred million)	1,292	108.7	242.6	0.0	2248.9	35.1
Flow	1,292	0.535	13.786	−14.603	462.417	0.136
Raw return	1,292	−0.104	4.819	−43.323	14.760	0.038
MKT-Adj return	1,292	−1.047	7.412	−43.428	22.755	−0.737
CAPM-Adj return	1,292	−0.117	4.820	−43.332	14.753	−0.054
Raw return volatility	1,019	4.283	1.752	1.497	8.590	4.182
MKT-Adj return volatility	1,019	7.360	2.643	2.085	12.324	6.899
CAPM-Adj return volatility	1,019	4.283	1.752	1.497	8.591	4.181
Age	1,292	12.96	5.33	2.00	21.00	14.00
Remuneration rate (%)	1,292	1.48	0.35	0.67	2.00	1.64
**Panel C: Non-ESG funds**						

**Variable**	***N***	**Mean**	**Std. Dev**	**Min**	**Max**	**Median**

AUM (hundred million)	4,290	108.7	242.6	0.0	2248.9	35.1
Flow	4,290	0.535	13.786	−14.603	462.417	0.136
Raw return	4,290	−0.104	4.819	−43.323	14.760	0.038
MKT-Adj return	4,290	−1.047	7.412	−43.428	22.755	−0.737
CAPM-Adj return	4,290	−0.117	4.820	−43.332	14.753	−0.054
Raw return volatility	3,502	4.283	1.752	1.497	8.590	4.182
MKT-Adj return volatility	3,502	7.360	2.643	2.085	12.324	6.899
CAPM-Adj return volatility	3,502	4.283	1.752	1.497	8.591	4.181
Age	4,290	12.96	5.33	2.00	21.00	14.00
Remuneration rate (%)	4,290	1.48	0.35	0.67	2.00	1.64

*In Table 1, Asset Under Management (AUM) is fund size; Flow, fund flow; Raw return, the average of returns over the previous 12 months; MKT-Adj return, the average of market-adjusted return over the previous 12 months; CAPM-Adj return, the average of CAPM-adjusted returns over the previous 12 months; Raw return volatility, the volatility of raw returns over the previous 12 months; MKT-Adj return volatility, the volatility of market-adjusted returns over the previous 12 months; CAPM-Adj return volatility, the volatility of CAPM-adjusted returns over the previous 12 months; Age, the history of the fund shown by the number of years; and Remuneration rate.*

**FIGURE 2:**
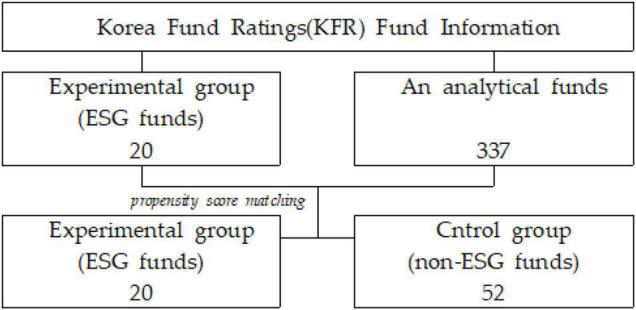
The sampling process.

Of all the funds since 2015, 52 non-ESG funds had similar characteristics to those of the experimental group. The ESG funds were matched to extract them as a control group. The extraction process is presented in Figure 2.

### Empirical Model

#### Measurement of Performance Benchmarks

The funds used in the analysis are samples of a minimum of 12 months of performance data. The estimation is made using the average of the 12-month return of the fund for the past 12 months (*Return*_*[t–1,t–12]*_), the average of the market-adjusted return ($r_{t}-r_{t}^{m}$), and the average of the CAPM-adjusted return.


(1) $$r_{t}-r_{f,t}=\alpha_{1}+\beta_{M\,K\,T}\,\left(r_{t}^{m}-r_{f,t}\right)+\in_{t}$$

Where: *r*_*t*_ is the monthly return of the fund; $r_{t}^{m}$ is the market-adjusted return during the period; and *r*_*f,  t*_ is the risk-free rate.

#### Method of Matching Non-Environmental, Social and Corporate Governance Funds With Environmental, Social and Governance Funds

To extract a control group under conditions similar to the experimental group, the propensity score matching methodology, proposed by Rosenbaum and Rubin (1983), was utilized. The propensity score of the variables that may affect business performance was calculated with a Probit Regression for 1:1 matching. The control group had a propensity score closest to that of the experimental group (Nearest Neighbor Matching).

To match the non-ESG funds with similar characteristics to those of the Korean ESG funds, the score is calculated based on the year of the fund, the size of the assets operated, and one CAPM factor. The three non-ESG funds having the smallest difference in the score were selected for the individual ESG fund.


(2) $$S\,c\,o\,r\,e\,{\left(a\right)}_{i,j}=\frac{{\left(A\,U\,M_{i}-A\,U\,M_{j}\right)}^{2}}{\sigma_{A\,U\,M}^{2}}+\frac{{\left(\beta_{i}-A\,U\,M_{j}\right)}^{2}}{\sigma_{A\,U\,M}^{2}}$$

Where Asset Under Management (AUM) is the size of the assets operated by the fund at the end of Month *t*; *i* refers to the ESG fund; and *j* refers to the non-ESG fund.

#### Fund Flows

According to the previous studies, there are various definitions of fund flow and methods for measuring that. In this study, the net change in fund assets is defined as fund flows. The fund flow that reflects money inflow and outflow is calculated using Eq. (3) (Sirri and Tufano, 1998).


(3) $$F\,l\,o\,w_{i,j}=\frac{A\,U\,M_{i,t}-A\,U\,M_{i,t-1}\,\left(1+r_{i,t}\right)}{\sigma_{A\,U\,M}^{2}}$$

#### Analysis Model for the Determinants of Fund Flow

A comparison of ESG fund investors with non-ESG fund investors to determine whether or not they had a relatively weaker fund flow–investment performance sensitivity was assumed through the regression equation in Eq. (4).


(4) $$Flow_{i,t}=\gamma_{0}+\beta_{1}Return_{i,\left[t-1,t-12\right]}+\beta_{2}Return_{i,\left[t-1,t-12\right]}\times E\,S\,G_{i}+\gamma_{1}\,C\,o\,n\,t\,r\,o\,l\,s_{i,t-1}+u_{i,t}$$

Where, *Flow*_*i,t*_ is money flows in Month *t* of Fund *i* measured with Eq. (3) above presented; *Return*_*i,  [t–1,  t–12]*_ is the average of the monthly rate of return of Fund *i* from *t-1* month through *t-12* months (or that of market-adjusted return or CAPM-adjusted return); dummy variable is indicated as $\{\begin{array}{c} if\;ESG\;fund=1\\ otherwise=0 \end{array}$; and Controls are measured with the fund characteristics, including size, age, fee, volatility of the rate of return, and front-end fee dummy as the major control variables. It was assumed that fund investors decide fund investment, considering other factors, such as the fund size, history, fee structure, fund performance volatility, and seller/operator reputation risk in addition to the past rate of return of the fund.

To analyze whether or not ESG funds are less sensitive to negative returns than non-ESG funds, we conducted the regression in Eq. (5).


(5) $$F\,l\,o\,w_{i,t}=\gamma_{0}+\left(\beta_{1}\,R^{+}+\beta_{2}\,R^{-}\right)\,R\,e\,t\,u\,r\,n_{i,\left[t-1,t-12\right]}+\left(\beta_{3}\,R^{+}+\beta_{24}\,R^{-}\right)\,R\,e\,t\,u\,r\,n_{i,\left[t-1,t-12\right]}\times E\,S\,G_{i}+\gamma_{1}\,C\,o\,n\,t\,r\,o\,l\,s_{i,t-1}+u_{i,t}$$

Where: *R*^+^ and *R*^−^ are dummy variables having a value of 1 if the monthly rate of return of the fund (or market-adjusted return or CAPM-adjusted return) is a positive (+) value or a negative (−) value, respectively. In Eq. (5), regression coefficients β_*1*_ and β_*2*_ represent the sensitivity of the fund flow to the positive (+) average raw return in comparison with that of the previous year and the sensitivity of the fund flow to the negative (−) average raw return for non-ESG fund, respectively. The regression coefficients β_*3*_ and β_*4*_ represent the sensitivity of the fund flow to the positive (+) average raw return and the sensitivity of the fund flow to the negative (−) average raw return for ESG funds, respectively.

The correlation between the fund performance volatility and fund flow is analyzed in Eq. (6).


(6) $$Flow_{i,t}=\gamma_{0}+\beta_{1}Vol_{i,\left[t-1,t-12\right]}+\beta_{2}Vol_{i,\left[t-1,t-12\right]}\times E\,S\,G_{i}+\gamma_{1}\,C\,o\,n\,t\,r\,o\,l\,s_{i,t-1}+u_{i,t}$$

Where: *Vol*_*i*, [*t*–1, *t*–12]_ represents the standard deviation of the monthly rate of return of Fund *i* from month *t-1* through months *t-12* (or the market-adjusted return or CAPM-adjusted return). Finally, according to the fund’s past performance, the difference between the asymmetry of the sensitivity between the fund performance volatility and the fund flow and the asymmetry of the sensitivity between the ESG funds and non-ESG funds is estimated in Eq. (7).


(7) $$F\,l\,o\,w_{i,t}=\gamma_{0}+\left(\beta_{1}\,R^{+}+\beta_{2}\,R^{-}\right)\,V\,o\,l_{i,\left[t-1,t-12\right]}+u_{i,t}$$

Where regression coefficients β_*1*_ and β_*2*_ represent the fund flow sensitivity to the volatility of the rate of return of non-ESG funds that realized a positive (+) average raw return in comparison with that of the previous year and the fund flow sensitivity to the volatility of the rate of return of the non-ESG funds that realized a negative (−) average raw return, respectively.

## Results of Empirical Analysis

The empirical analysis yielded several important results. First, the whole fund flow–performance sensitivity was analyzed. This included investigating whether or not ESG fund investors had weaker fund flow–performance sensitivity than non-ESG fund investors. Eq. (4) was used to test this; the results are presented in Table 2. Here, Panel A measured the fund performance with the average raw return over the previous 12 months and analyzed the fund flow–performance sensitivity. The entire sample had a negative (−) correlation between fund flow and fund performance. In other words, the lower the fund performance, the more the monetary outflow of the fund. The lower samples were analyzed by dividing the funds into the ESG fund and the non-ESG fund. The results show the same negative (−) sensitivity. In addition, no big difference in the sensitivity existed. Panels B and C examined the sensitivity between the fund flow and fund performance measured with the market-adjusted return and CAPM-adjusted return. This also had a negative (−) correlation, contrary to the results of previous studies showing a positive (+) correlation between fund flow and fund performance.

**TABLE 2:** Fund flow–performance sensitivity comparison.

CL.	All funds	ESG funds	Non-ESG funds
**Panel A**			
Raw return	−0.970*** (−54.42)	−0.963*** (−12.84)	−0.972*** (−129.31)
*N*	5,582	1,292	4,290
*R* ^2^	0.35	0.11	0.80
*F*	2,961.362	164.827	16,722.197
**Panel B**			
MKT-Adj return	−0.401*** (−29.86)	−0.371*** (−7.30)	−0.409*** (−45.63)
*N*	5,582	1,292	4,290
*R* ^2^	0.14	0.04	0.33
*F*	891.529	53.341	2,082.368
**Panel C**			
CAPM-Adj return	−0.969*** (−54.42)	−0.963*** (−12.84)	−0.972*** (−129.32)
*N*	5,582	1,292	4,290
*R* ^2^	0.35	0.11	0.80
*F*	2,961.359	164.827	16,722.419

Table 2 is the result of comparing the fund flow–performance sensitivity. The dependent variable is fund flow, Raw return is the average of returns over the previous 12 months; MKT-Adj return, the market-adjusted returns over the previous 12 months; CAPM-Adj return, CAPM-adjusted return over the previous 12 months. The content of the table is the regression coefficient value; the figure in parentheses, t-value; and ***, **, and * means that each is significant, respectively, at 1, 5, and 10%.

A regression equation was used to test Hypothesis 1. A dummy variable was added to show whether or not it is an ESG fund. The regression equation included return rate volatility and fund characteristics. The results are presented in Table 3. Models (1) and (2) used the raw return. Models (3) and (4) used the Market-Adjusted Return as the fund performance. Models (5) and (6) measured the CAPM-Adjusted Return as the fund performance. There was a significant negative (−) correlation found between the fund flow and the fund performance. However, the ESG fund did not affect the fund flow, unlike what was expected. This result dismisses Hypothesis 1 and is consistent with the results in Wee et al. (2020). In other words, the monetary flow is not affected by whether Korean funds are set up as an ESG or not (Wee et al., 2020).

**TABLE 3:** Fund flow–fund characteristics sensitivity.

CL.	Model 1	Model 2	Model 3	Model 4	Model 5	Model 6
Raw return	−0.971*** (−48.21)	−0.992*** (−381.82)				
Raw return×ESG	0.007 (0.16)	0.001 (0.16)				
Raw return volatility		0.025*** (3.99)				
MKT-Adj_Return			−0.408*** (−26.90)	−0.428*** (−50.99)		
MKT-Adj_Return×ESG			0.034 (1.06)	0.017 (0.96)		
MKT-Adj return volatility				−0.038** (−2.09)		
CAPM-Adj return					−0.971*** (−48.21)	−0.992*** (−381.80)
CAPM-Adj return×ESG					0.007 (0.16)	0.001 (0.17)
CAPM-Adj return volatility						0.026*** (4.04)
Size		−0.006 (−1.33)		0.004 (0.18)		−0.006 (−1.33)
Age		−0.006* (−1.81)		0.020 (1.27)		−0.006* (−1.85)
Remuneration rate		0.025 (0.63)		−0.054 (−0.28)		0.025 (0.64)
Fee dummy		−0.013 (−0.55)		0.015 (0.12)		−0.013 (−0.56)
*N*	5,582	4,521	5,582	4,521	5,582	4,521
*R* ^2^	0.35	0.98	0.14	0.42	0.35	0.98
*F*	1,480.435	26,284.837	446.335	467.362	1,480.433	26,281.104

Table 3 is the result of regression (OLS) analysis on the sensitivity of fund flow–fund characteristics. The dependent variable is fund flow. Raw return is the average of returns over the previous 12 months; MKT-Adj return, the market-adjusted returns over the previous 12 months; CAPM-Adj return, CAPM-adjusted return over the previous 12 months; Raw return volatility, the volatility of raw return over the previous 12 months; MKT-Adj return volatility, the volatility of the market-adjusted returns over the previous 12 months; CAPM-Adj return volatility, the volatility of CAPM-adjusted returns over the previous 12 months; ESG, a dummy that shows whether it is an ESG fund; Size, the natural logarithm of the size of the assets operated by the fund; Age, the age; Remuneration rate, the natural logarithm of a fee; and Fee dummy, the front-end fee dummy. The content of the table is the regression coefficient value; the figure in parentheses, t-value; and ***, **, and * means that each is significant, respectively, at 1, 5, and 10%.

Fund volatility had significant positive (+) impacts on the dependent variable in Model (2), where fund volatility was measured by the raw return volatility. Model (6) measured fund volatility with the CAPM-adjusted return volatility. It had a significant negative (−) impact in Model (4), which measured it with a market-adjusted return. In other words, the raw return and CAPM-adjusted return result in an increase in fund flow with larger volatility. The market-adjusted return yields an increase in fund flow with smaller volatility. This result suggests that fund flow–performance sensitivity may differ depending on the method used to measure fund performance.

We applied Eq. (5) to test Hypothesis 2. Hypothesis 2 focused on whether or not the sensitivity between the fund flow and fund performance would be asymmetric, according to the type of the fund’s past performance (i.e., positive and negative). This asymmetric sensitivity would also be weaker in ESG funds than in non-ESG funds. The results are summarized in Table 4. It was determined that a positive fund performance yields a (non)significant positive (+) fund flow–performance sensitivity, while a negative fund performance yields a (non)significant negative (−) fund flow–performance sensitivity. For Korean funds, the interaction between fund flow and fund performance was found to be significantly negative (−), regardless of fund performance, contrary to the expectation.

**TABLE 4:** Fund flow–performance sensitivity according to the type of past fund performance.

CL.	Model 1	Model 2	Model 3	Model 4	Model 5	Model 6
Raw return×R^+^	−0.964** (−29.05)	−0.991*** (−230.40)				
Raw return×R^–^	−0.978*** (−30.26)	−0.993*** (−229.18)				
Raw return×R^+^ ×ESG	0.016 (0.25)	−0.021** (−2.01)				
Raw return×R^+^ ×ESG	0.000 (0.00)	0.018** (2.00)				
Raw return volatility		0.041*** (4.58)				
MKT-Adj_Return×R^+^			−0.323*** (−11.51)	−0.382*** (−23.80)		
MKT-Adj_Return×R^–^			−0.471*** (−20.84)	−0.461*** (−35.87)		
MKT-Adj_Return×R^+^ ×ESG			0.089* (1.67)	0.158*** (4.44)		
MKT-Adj_Return×R^–^ ×ESG			0.009 (0.23)	−0.071*** (−2.65)		
MKT-Adj return volatility				−0.114*** (−4.60)		
CAPM-Adj return×R^+^					−0.964*** (−29.01)	−0.990*** (−230.10)
CAPM-Adj return×R^–^					−0.978*** (−30.30)	−0.993*** (−229.44)
CAPM-Adj return×R^+^ ×ESG					0.016 (0.25)	−0.022** (−2.03)
CAPM-Adj return×R^–^ ×ESG					−0.000 (−0.00)	0.018** (2.03)
CAPM-Adj return volatility						0.041*** (4.64)
Size		−0.006 (−1.37)		0.011 (0.49)		−0.006 (−1.36)
Age		−0.006* (−1.83)		0.021 (1.33)		−0.006* (−1.87)
Remuneration rate		0.026 (0.66)		−0.070 (−0.36)		0.026 (0.66)
Fee dummy		−0.014 (−0.57)		0.016 (0.13)		−0.014 (−0.58)
*N*	5,582	4,521	5,582	4,521	5,582	4,521
*R* ^2^	0.35	0.98	0.14	0.43	0.35	0.98
*F*	740.011	20,465.331	228.930	373.756	740.011	20,462.784

Table 4 is the result of regression analysis (OLS) of fund flow–performance sensitivity according to the type of past fund performance. The dependent variable is fund flow. Raw return is the average of returns over the previous 12 months; MKT-Adj return, the market-adjusted returns over the previous 12 months; CAPM-Adj return, CAPM-adjusted return over the previous 12 months; Raw return volatility, the volatility of raw return over the previous 12 months; MKT-Adj return volatility, the volatility of the market-adjusted returns over the previous 12 months; CAPM-Adj return volatility, the volatility of CAPM-adjusted returns over the previous 12 months; ESG, a dummy that shows whether it is an ESG fund; Size, the natural logarithm of the size of the assets operated by the fund; Age, the age; Remuneration rate, the natural logarithm of a fee; and Fee dummy, the front-end fee dummy. The content of the table is the regression coefficient value; the figure in parentheses, t-value; and ***, **, and * means that each is significant, respectively, at 1, 5, and 10%.

In Models (2) and (6), we examined the interactions between raw return and CAPM-adjusted return by including a dummy variable ESG. The results reveal a negative (−) impact on the dependent variable when it had a positive (+) value and a significantly positive (+) impact when it had a negative (−) value. This means that when the fund’s past performance is positive, the fund flow sensitivity decreases in the ESG funds. When it is negative, the sensitivity increases. We examined the interaction between the market-adjusted return and the ESG dummy variable in Models (3) and (4). In reverse, when it had a positive (+) value, there was a significantly positive (+) impact on the dependent variable. When it had a negative (−) value, there was a significantly negative (−) impact. Thus, the results are inconsistent.

In summary, the ESG fund shows asymmetric sensitivity between fund flow and fund performance according to the type of the fund’s past performance. With a positive fund performance, the ESG fund sensitivity is much higher (Bollen, 2007). In addition, in both cases, there is a difference in the sensitivity between the ESG and non-ESG funds.

Table 5 presents the results of an analysis conducted on the difference in the sensitivity between fund flow and performance volatility between the ESG and non-ESGs funds using Eq. (6) Hypothesis 3. The results reveal that regardless of the method for measuring past performance and whether it is an ESG fund or not, fund performance volatility and fund flow had a negative (−) correlation. Hence, the lower the investors’ fund performance volatility, the lower their awareness of the risk becomes, and the higher their expected performance becomes. Thus, they postpone the act of liquidating the fund, and the monetary outflow decreases.

**TABLE 5:** Comparison of the fund flow–performance volatility sensitivity.

CL	All funds		ESG funds		Non-ESG funds	
**Panel A**						
Raw return volatility	−0.441*** (−12.83)	−0.992*** (−420.22)	−0.730*** (−8.05)	−0.994*** (−96.22)	−0.395*** (−10.61)	−0.992*** (−907.57)
Raw return		0.010* (1.87)		0.065** (2.17)		0.002 (0.79)
*N*	4,521	4,521	1,019	1,019	3,502	3,502
*R* ^2^	0.04	0.98	0.06	0.91	0.03	1.00
*F*	164.642	91,591.089	64.744	4,956.603	112.577	425,144.247
**Panel B**						
MKT-Adj return volatility	−0.222*** (−8.38)	−0.422*** (−57.14)	−0.291*** (−4.74)	−0.405*** (−24.83)	−0.207*** (−7.03)	−0.426*** (−51.55)
MKT-Adj_Return		−0.173*** (−8.56)		−0.232*** (−4.78)		−0.160*** (−7.21)
*N*	4,521	4,521	1,019	1,019	3,502	3,502
*R* ^2^	0.02	0.43	0.02	0.39	0.01	0.44
*F*	70.288	1,693.052	22.448	326.240	49.465	1,372.208
**Panel C**						
CAPM-Adj return volatility	−0.441*** (−12.83)	−0.992*** (−420.18)	−0.730*** (−8.05)	−0.994*** (−96.21)	−0.395*** (−10.61)	−0.992*** (−907.95)
CAPM-Adj return		0.011** (2.03)		0.066** (2.23)		0.003 (1.10)
N	4,521	4,521	1,019	1,019	3,502	3,502
R^2^	0.04	0.98	0.06	0.91	0.03	1.00
F	164.653	91,576.132	64.745	4,955.576	112.584	42,549.066

Table 5 is the result of comparing fund flow–performance volatility sensitivity. The dependent variable is fund flow. Raw return is the average of returns over the previous 12 months; MKT-Adj return, the market-adjusted returns over the previous 12 months; CAPM-Adj return, CAPM-adjusted return over the previous 12 months; Raw return volatility, the volatility of raw return over the previous 12 months; MKT-Adj return volatility, the volatility of the market-adjusted returns over the previous 12 months; CAPM-Adj return volatility, the volatility of CAPM-adjusted returns over the previous 12 months; The content of the table is the regression coefficient value; the figure in parentheses, t-value; and ***, **, and * means that each is significant, respectively, at 1, 5, and 10%.

The previous analysis showed a similar result when the interaction variable between performance volatility and the ESG dummy was added to the entire sample. In Table 6, to examine the result of the analysis of Models (1), (2), (5), and (6) in the ESG funds, it is noted that there is a negative (−) interaction between performance volatility and fund flow. This is a result following the positive feedback trading according to the financial factors, which is quite different from the ground for the original hypothesis setting. Consequently, Hypothesis 3 was dismissed.

**TABLE 6:** Fund flow–performance volatility sensitivity.

CL.	Model 1	Model 2	Model 3	Model 4	Model 5	Model 6
Raw return volatility	−0.429*** (−12.32)	−0.451*** (−12.64)				
Raw return volatility×ESG	−0.085** (−2.16)	−0.104*** (−2.60)				
MKT-Adj return volatility			−0.216*** (−8.06)	−0.222*** (−8.14)		
MKT-Adj return volatility×ESG			−0.031 (−1.30)	−0.035 (−1.46)		
CAPM-Adj return volatility					−0.429*** (−12.32)	−0.451*** (−12.64)
CAPM-Adj return volatility×ESG					−0.085** (−2.16)	−0.104*** (−2.60)
Size		−0.080*** (−2.74)		−0.038 (−1.28)		−0.080*** (−2.74)
Age		−0.009 (−0.44)		0.002 (0.07)		−0.009 (−0.44)
Remuneration rate		0.062 (0.25)		−0.052 (−0.21)		0.062 (0.25)
Fee dummy		−0.021 (−0.13)		−0.005 (−0.03)		−0.021 (−0.13)
*N*	4,521	4,521	4,521	4,521	4,521	4,521
*R* ^2^	0.04	0.04	0.02	0.02	0.04	0.04
*F*	84.721	29.637	35.991	12.289	84.726	29.639

Table 6 is the result of analyzing fund flow–performance volatility sensitivity. The dependent variable is fund flow. Raw return volatility, the volatility of raw return over the previous 12 months; MKT-Adj return volatility, the volatility of the market-adjusted returns over the previous 12 months; CAPM-Adj return volatility, the volatility of CAPM-adjusted returns over the previous 12 months; ESG, a dummy that shows whether it is an ESG fund; Size, the natural logarithm of the size of the assets operated by the fund; Age, the age; Remuneration Rate, the natural logarithm of a fee; and Fee Dummy, the front-end fee dummy. The content of the table is the regression coefficient value; the figure in parentheses, t-value; and ***, **, and * means that each is significant, respectively, at 1, 5, and 10%.

It turned out that, of the variables representing the fund properties, the size variable was found to negatively affect the dependent variable. This means that the smaller the fund size, the lower the fund flow sensitivity becomes.

The result of the Hypothesis 4 test on the difference in volatility according to fund flow sensitivity and the quality of fund performance is summarized in Table 7. The analysis revealed that the positive fund’s past performance consistently had a negative (−) impact on the fund flow, while the negative past performance had a positive (+) impact on it in all samples of Panels A–C. Since the expected performance is high when the past performance of a fund is positive, fund flow sensitivity is low. Since the expected performance is low when the fund’s past performance is negative, the sensitivity is high. This result partially supports Hypothesis 4. Therefore, the sensitivity between fund flow and performance volatility is asymmetric according to the quality of the fund’s performance. However, there is little difference between the ESG fund sensitivity asymmetry and the non-ESG fund sensitivity asymmetry.

**TABLE 7:** Comparison of fund flow–performance volatility sensitivity according to the type of past fund performance.

CL.	All funds	ESG funds	Non-ESG funds
**Panel A**			
Raw return volatility×R^+^	−0.919*** (−33.42)	−0.938*** (−13.48)	−0.910*** (−30.19)
Raw return volatility×R^–^	0.486*** (15.77)	0.543*** (6.50)	0.475*** (14.31)
*N*	4,521	1,019	3,502
*R* ^2^	0.44	0.45	0.44
*F*	1,779.480	422.831	1,358.093
**Panel B**			
MKT-Adj return volatility×R^+^	−0.531*** (−26.53)	−0.540*** (−11.75)	−0.529*** (−23.76)
MKT-Adj return volatility×R^–^	0.371*** (17.20)	0.367*** (7.27)	0.372*** (15.58)
*N*	4,521	1,019	3,502
*R* ^2^	0.47	0.47	0.47
*F*	2,020.943	449.263	1,570.108
**Panel C**			
CAPM-Adj return volatility×R^+^	−0.918*** (−33.39)	−0.936*** (−13.45)	−0.910*** (−30.17)
CAPM-Adj return volatility×R^–^	0.486*** (15.79)	0.545*** (6.52)	0.470*** (14.32)
*N*	4,521	1,019	3,502
*R* ^2^	0.44	0.45	0.44
*F*	1,778.305	422.448	1,357.286

Table 7 is the result of comparing the fund flow–performance volatility sensitivity according to the type of past fund performance. The dependent variable is fund flow. Raw return is the average of returns over the previous 12 months; MKT-Adj return, the market-adjusted returns over the previous 12 months; CAPM-Adj return, CAPM-adjusted return over the previous 12 months; is the positive value for the raw return, MKT-Adj return, CAPM-Adj return, respectively, is the negative value for the raw return, MKT-Adj return, CAPM-Adj return, respectively, The content of the table is the regression coefficient value; the figure in parentheses, t-value; and ***, **, and * means that each is significant, respectively, at 1, 5, and 10%.

## Conclusion

Rational investors make investments based on risk appetite and the fund’s past performance. In other words, they expect a positive (+) correlation between the fund’s previous returns and the fund flow. However, ESG fund investors reflect upon other non-financial factors (e.g., the purpose of the fund investment, size, age, return volatility, and fee structure), rather than just on the fund’s previous returns. Thus, it is expected that ESG fund investors would have weaker fund performance–fund flow sensitivity than non-ESG fund investors.

This study analyzed the sensitivity between Korean fund flow and fund performance. We examined whether there were differences in the sensitivity between ESG funds and non-ESG funds. We also examined the sensitivity according to the type of fund’s past performance (i.e., positive and negative). In addition, this study analyzed the correlation between the fund flow–performance volatility to check the fund flow–past performance interaction and asymmetry and the difference in the sensitivity between ESG and non-ESG funds.

The primary results of the empirical analysis are as follows. First, it is noted that there is a negative (−) correlation between Korean fund flow and fund performance. Furthermore, unlike what was expected, in ESG funds, there was no impact on fund flow. This implies that fund flow is more sensitive when Korean funds have a poor return. Whether it is ESG or not does not affect the fund flow. On the other hand, the fund volatility showed different sensitivity according to the method used to measure the fund’s performance.

Second, the analysis of the asymmetry between fund flow and performance sensitivity, according to the type of the fund’s past performance, revealed that the fund flow–performance interaction had a negative (−) correlation. This was the case regardless of the quality of the fund’s performance. This result was not expected. Hence, the interaction between the fund’s past performance and the ESG dummy variable yielded asymmetric sensitivity. The sensitivity of the ESG funds was a little higher when the performance was positive. In the meantime, the analysis of the difference in the sensitivity between fund flow and fund performance volatility, measured by dividing the funds into ESG and non-ESG funds, revealed that fund flow and fund performance volatility had a negative (−) correlation, regardless of the method used for measuring fund performance and whether or not it was an ESG fund. Therefore, the lower the investors’ fund performance volatility, the lower their risk awareness and the higher their expected performance. As a result, the less the monetary outflow of the fund becomes. The analysis of the entire sample with the interaction variable between the fund performance volatility and the ESG dummy added yielded negative (−) interactions with fund flow. This result shows that investors are more sensitive to the price than to the purpose of the ESG investment.

Finally, the comparison of fund flow–performance volatility sensitivity according to the type of fund’s past performance reveals that it is consistently asymmetric, according to the quality of the fund’s performance. However, it is noted that there is almost no difference between the ESG fund sensitivity asymmetry and the non-ESG fund sensitivity asymmetry.

The results deviate from the previous expectations that Korean ESG fund investors would focus more on non-financial properties (e.g., the purpose of investment) than on returns. The general investors’ choice of ESG fund is to choose nice companies with good performance, instead of simply investing in good companies. Thus, ESG fund sellers and operators should not overlook the fact that ESG fund investors also attach importance to fund performance. Hence, they should attract investors through developing various ESG fund products that can continue to create and maintain high performance. This means that the screening of the environment, governance, and social responsibility has not been working when investors choose a fund. This proves that there is still a lack of awareness of ESG despite that receives global attention.

ESG fund selling and management companies to develop ESG fund products that can maintain and create a high performance without overlooking the fact that ESG fund investors, of course, attach importance to fund performance as well in the early stages of ESG funds. However, it would be necessary to induce investors to use related screening as a factor supplementing financial performance by making them more interested in social agendas such as the environment (climate change), fair society, shared growth, ethics, and morality. Since the differing effect of capital inflow according to the type of screening is a kind of the clientele effect, it is necessary to develop ESG fund products with a variety of screening.

It would be necessary to examine the relationship between the ESG fund performance and the fund flow once again in the future and check if there is any change if the data are accumulated as ESG funds become more generalized after the awareness of ESG grows further, and investors become more interested in the practical properties of ESG funds.

## Data Availability Statement

The original contributions presented in the study are included in the article/supplementary material, further inquiries can be directed to the corresponding author/s.

## Author Contributions

DK: conceptualization and investigation. YK: data curation and resources. IK: formal analysis, writing – original draft, and methodology. DK and YK: writing – review and editing. All authors have read and agreed to the published version of the manuscript.

## Conflict of Interest

The authors declare that the research was conducted in the absence of any commercial or financial relationships that could be construed as a potential conflict of interest.

## Publisher’s Note

All claims expressed in this article are solely those of the authors and do not necessarily represent those of their affiliated organizations, or those of the publisher, the editors and the reviewers. Any product that may be evaluated in this article, or claim that may be made by its manufacturer, is not guaranteed or endorsed by the publisher.

## Footnotes

1 An ESG fund is issued to raise the fund related to Socially Responsible Investment (SRI) such as Environmental, Social, Governance improvement.

2 ESG and financial performance aggregated evidence from more than 2,000 empirical studies (2015.9).

## References

[B1] Berk J. Green R. (2004). Mutual fund flows and performance in rational markets. *J. Polit. Econ.* 112 1269–1295. 10.1086/424739

[B2] Black F. (1996). Noise. *J. Finance* 41 529–543.

[B3] Bollen N. (2007). Mutual fund attributes and investor behavior. *J. Financ. Quant. Anal.* 42 683–708. 10.1017/s0022109000004142

[B4] Busse J. (1999). Volatility timing in mutual funds: evidence from daily returns. *Rev. Financ. Stud.* 12 1009–1041.

[B5] Chevalier J. Ellison G. (1997). Risk taking by mutual funds as a response to incentives. *J. Polit. Econ.* 105 1167–1200.

[B6] Del Guercio D. Tkac P. (2002). The determinants of the flow of funds of managed portfolios: mutual funds vs. pension funds. *J. Financ. Quant. Anal.* 37 523–557. 10.2307/3595011

[B7] Friede G. Busch T. Bassen A. (2015). ESG and financial performance: aggregated evidence from more than 2000 empirical studies. *J. Sustain. Finance Invest.* 5 210–233. 10.1080/20430795.2015.1118917

[B8] Gruber M. (1996). Another puzzle: the growth in actively managed mutual funds. *J. Finance* 51 783–810. 10.1111/j.1540-6261.1996.tb02707.x

[B9] Ippolito R. (1992). Consumer reaction to measures of poor quality: evidence from the mutual fund industry. *J. Law Econ.* 35 45–70. 10.1086/467244

[B10] James C. Karceski J. (2006). Investor monitoring and differences in mutual fund performance. *J. Bank. Finance* 30 2787–2808.

[B11] Korea Fund Ratings [KFR] (2021). *KFR Korea Fund Evaluation.* Available online at: www.kfr.co.kr

[B12] Lee C. Shleifer A. Thaler R. (1991). Investor sentiment and the closed-end fund puzzle. *J. Finance.* 46 75–109.

[B13] Morgan Stanley (2019). *Sustainable Signals: Growth and Opportunity in Asset Management.* New York, NY: Morgan Stanley Institute for Sustainable Investing.

[B14] Renneboog L. Horst J. Zhang C. (2011). Is ethical money financially smart? Nonfinancial attributes and money flows of socially responsible investment funds. *J. Financ. Intermed.* 20 562–588.

[B15] Rosenbaum P. R. Rubin D. B. (1983). The central role of the propensity score in observational studies for causal effects. *Biometrilca.* 70 41–55.

[B16] Sirri E. Tufano P. (1998). Costly search and mutual fund flows. *J. Finance* 53 1589–1622.

[B17] Victor Hugo (2019). “Sustainable finance: nothing is as powerful as an idea whose time has come,” in *Global Financial Stability Report*, (Washington, DC: IMF), 81–92.

[B18] Wee K.-W. Kang Y.-S. Chung J. M. Lee J. H. (2020). The effect of ESG levels on fund performance and cash flows. *Financ. Plan. Rev.* 13 83–115. 10.36029/fpr.2020.05.13.2.83

